# Systemic-pulmonary arteriovenous fistulae with pulmonary hypertension: A case report

**DOI:** 10.1097/MD.0000000000009959

**Published:** 2018-02-23

**Authors:** Yan-Ping Shi, Yi-Dan Li, Xiu-Zhang Lv, Yuan-Hua Yang

**Affiliations:** a Department of Echocardiography; b Department of Respiratory, Beijing Chaoyang Hospital, Capital Medical University, Beijing, P.R. China.

**Keywords:** angiography, arteriovenous fistulae, echocardiography, pulmonary hypertension

## Abstract

**Background:**

Arteriovenous (AV) fistulae is an extremely rare disease of vascular malformation that involves fistulae formation between the systemic and pulmonary AV systems.

**Case representation:**

This case report describes a rare systemic-pulmonary AV fistulae of congenital origin, accompanied by pulmonary hypertension, as determined by aortic angiography and echocardiography.

**Conclusion:**

Characteristics, diagnosis, and therapeutic approaches of this rare abnormality are explored.

## 1 Introduction

Connections between the systemic and pulmonary systems are rare and may be congenital or acquired.^[1,2]^ Patients most often are asymptomatic but may show cyanosis, dyspnea, or hemoptysis.^[3]^ Systemic artery angiography is the gold standard for diagnosis, with surgery and interventional embolization being used as the primary treatment methods. Here, we report a patient with dyspnea who later received an unexpected diagnosis of systemic-pulmonary arteriovenous (AV) fistulae of congenital origin. Due to the patient's extensive lesions, his pulmonary pressure did not decrease to normal levels either immediately or by 1 year after treatment. Written informed consent for this case report was obtained from the patient.

## 2 Case report

The study was approved by the ethical committee of Beijing Chaoyang Hospital, Capital Medical University, China. Written informed consent was obtained.

A 32-year-old man presented with illness-related fever and unexplained dyspnea of 2 days’ duration. He was referred to the Department of Respiration at Beijing Chaoyang Hospital (Beijing, China) on April 20, 2015. Medical history was remarkable for giant cell tumor of the bone 15 years before, followed by replacement of the left femoral head 14 years before due to tumor progression. He reported a diagnosis of gout 7 years before. Routine physical examination on the day of admission revealed elevations in body temperature (38.3°C), heart rate (120 beats per minute), and respiratory frequency (30 times/min). He did not appear to have cyanosis or clubbing. Routine blood, biochemistry, urine, and blood coagulation tests revealed no remarkable findings. Arterial blood gas analysis showed that the patient had type I respiratory failure (pH 7.474, partial pressure of carbon dioxide [PCO_2_] 24.7 mm Hg, partial pressure of oxygen [PO_2_] 53.9 mm Hg, blood oxygen saturation [SpO_2_] 90.2%, concentration of bicarbonate ion [HCO_3_^-^] 18.4 mmol/L, and buffer excess [BE] −2.9 mmol/L). His N-terminal pro-brain natriuretic peptide (NT-pro BNP) level was 593 pg/mL on the second day after hospital admission.

**Figure 1:**
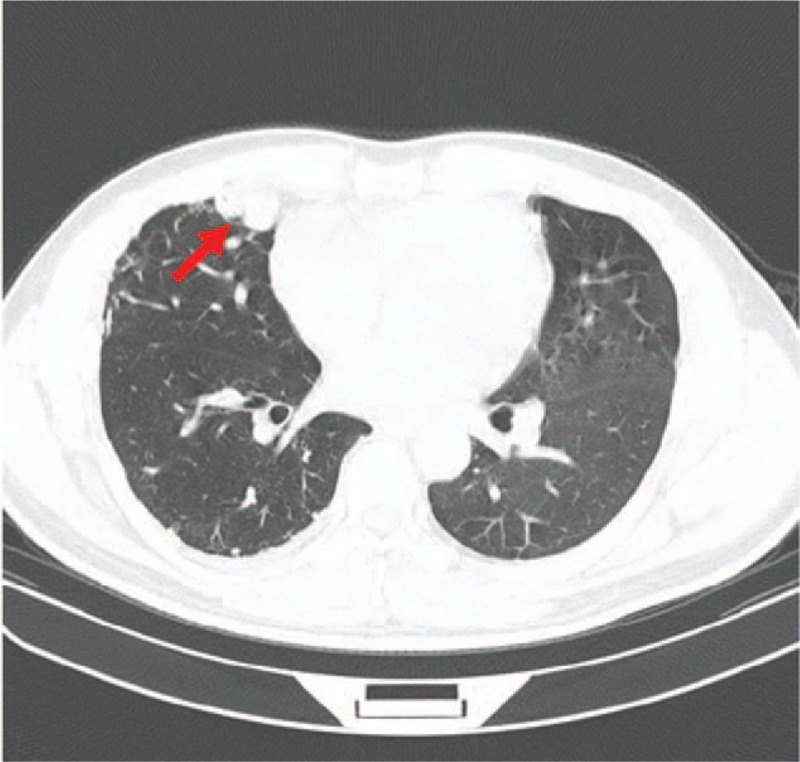
High-resolution CT showing an abnormal vascular mass (red arrow; ∼8.0 × 2.5 mm in size) in the medial segment of the middle lobe of the right lung near the pleura.

**Figure 2:**
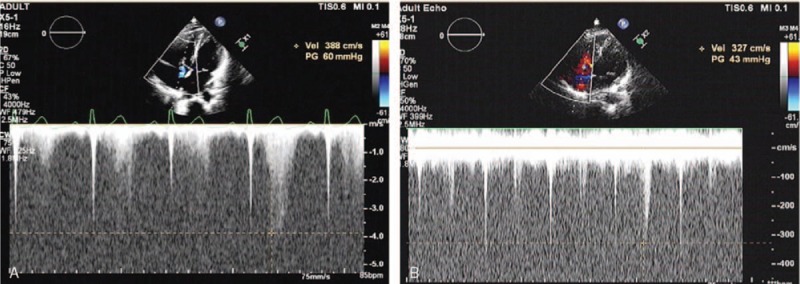
Echocardiography with the tricuspid insufficiency method showed that the systolic blood pressure of the pulmonary artery was ∼70 mm Hg in April 2015 (A) and May 2016 (B). CT = computed tomography.

Two days after admission, a pulmonary high-resolution computed tomography (CT) scan revealed a small amount of inflammation in the lower lobes of the both lungs and an abnormal vascular mass in the middle lobe of the right lung (Fig. 1). Echocardiography indicated that the patient had pulmonary hypertension (Fig. 2A) with right heart dysfunction. Fractional area change (FAC) of the right ventricular area was 21.8%, tricuspid annular plane systolic excursion (TAPSE) was 18.5 mm, and right ventricular index of myocardial performance (Tei index) was 0.93.

After antiviral and antibacterial treatment, the patient's temperature dropped to a normal range on April 27, 2015. Although his symptoms of asthma improved, blood oxygen saturation remained lower than normal (90.4%). Over the next week, several examinations were carried out, including D-dimer (0.36 mg/L FEU; ≤ 0.55 mg/L indicates a smaller likelihood of pulmonary embolism), immune-related tests, right heart contrast echocardiography, and systemic PET/CT due to pulmonary hypertension. No major abnormalities were found.

**Figure 3:**
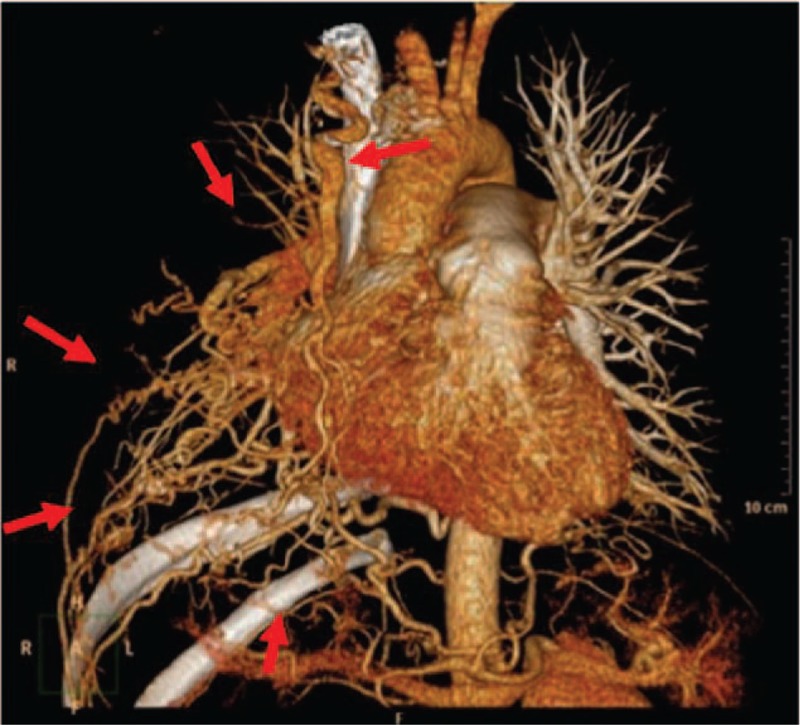
CT pulmonary angiography demonstrating that compared to the left pulmonary vessels, the right pulmonary arteries, intercostal arteries, and internal mammary artery were tortuous and dilated. Red arrows on the right side of the ascending aorta point to the internal mammary artery, and intercostal arteries are parallel to the ribs. Area around the red arrows is the range of the fistulae. CT = computed tomography.

To determine the cause of pulmonary hypertension, and considering the abnormal vascular findings of the right lung, the patient underwent imaging studies. Pulmonary ventilation/perfusion imaging showed no blood flow in the right lung. Pulmonary CT angiography revealed tortuous and dilated right pulmonary, intercostal, internal mammary, and phrenic arteries (Fig. 3). Aortic angiography on May 12, 2015 led to a diagnosis of congenital multiple systemic-pulmonary AV fistulae that resulted in pulmonary hypertension and hypoxia. Right cardiac catheterization was performed in response to the pulmonary hypertension (mean pulmonary pressure = 26 mm Hg).^[4]^

## 3 Discussion

Fistulae communication between the systemic and pulmonary arteries or pulmonary veins is rare. According to relevant case reports, disease onset is at a relatively young age. Most patients are asymptomatic or have hemoptysis as their first symptom, accompanied by varying degrees of chest tightness, heart palpitations, shortness of breath, cyanosis, and dyspnea. Lesions typically are located in a unilateral lung, often in the lower lobes. Most cases of anomalous systemic arteries originate from the descending aorta.^[4–6]^

We report a rare case of an adult patient with a wide range of fistulae, accompanied by pulmonary hypertension. The systemic circulation pressure was higher than the pulmonary circulation pressure. Because of the multiple pulmonary arteriovenous fistulae, there was systemic circulation of blood flow to the right lung, which led to low or no normal pulmonary blood flow perfusion in the lung. Right pulmonary systems accepted the abnormal arterial blood flow of the systemic arteries, which eventually resulted in pulmonary hypertension. Fistulae communication was detected by aortic angiography.

Although systemic artery angiography is the gold standard for diagnosis, other ancillary tests are essential. Our patient's condition was complex. We had to rule out each potential cause of pulmonary hypertension (e.g., connective tissue disorder, tumor, chronic thromboembolic disorder, and congenital heart disease) through a series of comprehensive examinations. Echocardiography results showing pulmonary hypertension provided a breakthrough for the final diagnosis of the disease.

**Figure 4:**
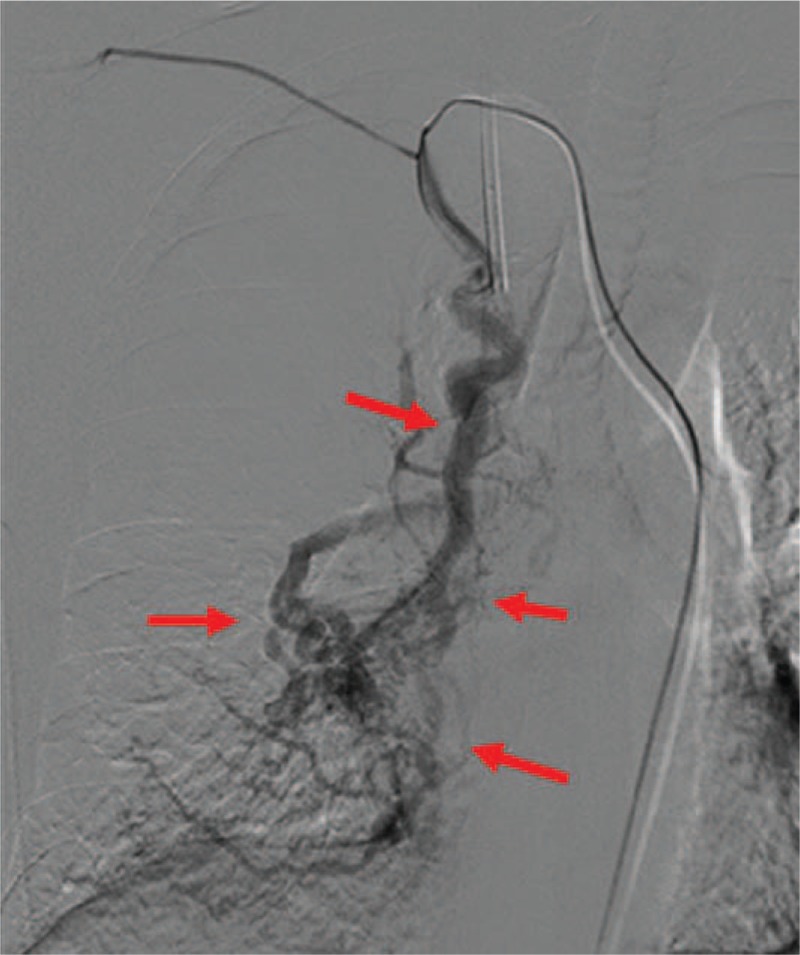
Aortic angiography revealed fistulae (red arrows show the range) between the internal mammary artery and pulmonary vein.

Surgical and interventional embolization therapy are effective treatments for this kind of disease. When the lesions are limited, surgical resection of the diseased pulmonary lobe or ligation of the anomalous artery is an acceptable choice. However, our patient had extensive lesions and unusual systemic arteries as feeding vessels of the AV fistulae, which precluded surgical treatment. During aortic angiography imaging for diagnosis, we used spring coils to embolize to the patient's right internal mammary artery (the largest abnormal artery) for treatment (Fig. 4). However, no change in pulmonary pressure was detected with the right heart catheter after surgery (mean pulmonary pressure = 28 mm Hg). This outcome was thought to be a result of abnormal vascular compensation. The patient was recommended to have regular follow-ups, and further embolization treatment was not suggested or planned.

The prognosis of congenital systemic-pulmonary AV fistulae is poor, and follow-up is important. After hospital discharge, the patient was seen and underwent echocardiography once every 6 months. Two follow-up echocardiographic examinations have been performed to date, in November 2015 and May 2016. In both cases, his pulmonary systolic pressure was ∼54 mm Hg (Fig. 2B), and his right heart function had returned to normal (FAC 39.9%, TAPSE 21.7 mm, Tei index 0.47). These outcomes may be related to the patient's age and exercise habits.

In summary, the present study reports a case of rare systemic-pulmonary AV fistulae of congenital origin, accompanied by pulmonary hypertension. Various factors were assessed for their potential role in causing pulmonary hypertension in this patient. This report serves not only as an example of a rare disease in a patient with pulmonary hypertension, but also points to the importance of adequate assessment according to the patient's unique situation.
